# Meaningful or Marginal: An Integrative Review of Consumer and Community Involvement in Migrant Health Research

**DOI:** 10.3390/healthcare14142065

**Published:** 2026-07-09

**Authors:** Mehwish Nisar, Muhammad Waqas Nisar Ahmed, Sobia Zafar

**Affiliations:** 1 School of Public Health, Faculty of Health, Medicine and Behavioural Sciences, The University of Queensland, Brisbane, QLD 4072, Australia; 2 Community Medicine Department, Jinnah Sindh Medical University, Karachi 75510, Pakistan; waqas.nisar@jsmu.edu.pk; 3 School of Dentistry, Faculty of Health, Medicine and Behavioural Sciences, The University of Queensland, Brisbane, QLD 4006, Australia; s.zafar@uq.edu.au

**Keywords:** patient and public involvement, consumer and community involvement, migrant health, health equity, research governance

## Abstract

**Highlights:**

**What are the main findings?**

- Consumer and Community Involvement in migrant health research appears more marginal than meaningful, with structural conditions tending to limit authentic participation from priority setting and study design through to governance and data decisions.
- Structural conditions, rather than individual failings, appear to skew participation toward English-speaking migrants of higher socioeconomic status, potentially underrepresenting the most vulnerable subgroups.

**What are the implications of the main findings?**

- Procedural compliance with involvement requirements appears insufficient; reform across research governance, funding structures, and ethics processes is warranted.
- Future research should prioritise developing and empirically evaluating intersectional recruitment frameworks and migrant-led co-design models that can reach the most marginalised migrant communities.

**Abstract:**

**Background/Objectives:** Consumer and Community Involvement (CCI) is increasingly mandated in health research to promote ethical, relevant, and impactful research outcomes. However, in migrant health research, CCI can sometimes be tokenistic. This review aimed to critically synthesise the empirical and contextual literature on CCI in migrant health research to examine how meaningfully migrant communities are engaged across the research process. **Methods:** An integrative literature review was conducted. A systematic search of six databases (CINAHL, EMBASE, PubMed, PsycINFO, Scopus, Web of Science) was performed for peer-reviewed literature published up to January 2026, excluding grey literature. Two independent reviewers conducted screening and quality appraisal (using AMSTAR 2, MMAT, and the JBI Critical Appraisal Checklist for Text and Opinion). Data from 20 included studies were synthesised using systematic text condensation. **Results:** A total of 20 peer reviewed articles were included. Results identified four themes: (1) the meaningfulness gap—between superficial indicators of involvement and impact-driven measures; (2) the burden of representation—few individuals expected to act as authentic representatives for heterogeneous populations; (3) intersectional power dynamics—participation skewed toward English-speaking, high-socioeconomic status migrants; and (4) limited co-design in research governance—insufficient involvement in funding, ethics, or data decisions. **Conclusions:** CCI in migrant health research appears more marginal than meaningful. Structural conditions, rather than individual failings, appear to constrain authentic community participation particularly at the stages of priority setting and research governance. Procedural compliance alone appears insufficient; reform across research governance, funding, and ethics appears warranted.

## 1. Introduction

Over recent decades, Consumer and Community Involvement (CCI) in health and medical research has undergone a fundamental transformation, evolving from a peripheral ethical aspiration to an institutionally mandated component of rigorous inquiry, particularly within high-income Western jurisdictions such as the United Kingdom (UK National Institute for Health and Care Research) and Australia (National Health and Medical Research Council) [1]. This shift reflects growing recognition that individuals with lived experience hold valuable experiential knowledge that complements and extends traditional academic and clinical expertise. Internationally operationalised as Patient and Public Involvement (PPI), CCI is defined as research conducted “with” or “by” consumers and community members, rather than “to,” “about,” or “for” them [2,3]. This distinction reframes individuals with lived experience as active partners who contribute to shaping research priorities, study design, implementation, and knowledge translation [4,5].

Despite these institutional mandates, the implementation of CCI remains uneven across populations. Migrant and minority communities, an umbrella term encompassing distinct legal and social categories such as culturally and linguistically diverse (CALD) populations, asylum seekers, undocumented migrants, and recently arrived refugees, are frequently underrepresented or excluded from health research and related CCI initiatives [6,7]. These populations often experience pronounced health inequities shaped by complex social determinants, including language barriers, insecure employment, systemic discrimination, and, in some cases, the trauma associated with forced displacement [8]. Importantly, these same structural factors also act as barriers to meaningful research participation [8].

When implemented in migrant health research, CCI often falls short of genuine partnership, frequently manifesting as tokenistic engagement. Drawing on Sherry Arnstein’s “Ladder of Citizen Participation,” tokenism occurs when marginalised groups are included primarily to fulfil institutional requirements, without meaningful influence over decision-making [9]. In the context of migrant health research, involvement is often limited to later stages of the research process, such as data collection or dissemination, rather than earlier phases of priority setting, co-design, and governance. This superficial engagement may not adequately address the power imbalances between academic institutions and vulnerable migrant populations [10].

These challenges are further compounded by the intersectional nature of migrant identities. Building on Kimberlé Crenshaw’s theory of intersectionality, overlapping social positions, such as race, gender, class, and migration status can intensify marginalisation [11]. In health research, intersectional power dynamics often result in a “burden of representation,” whereby a small group of more accessible or privileged migrants are repeatedly engaged to represent diverse communities [12]. This not only places undue strain on these individuals but may also limit the inclusion of more marginalised voices, including non-English speakers, undocumented individuals, and recently arrived migrants.

While previous systematic reviews have explored the broad impacts of PPI in general health research [13], and others have mapped participatory approaches in specific migrant health interventions [14], this review distinctly examines the stage of involvement, governance roles, and representational mechanisms specific to migrant population. Therefore, this integrative review aims to critically appraise and synthesise the empirical and contextual literature on CCI in migrant health research to examine the extent to which migrant communities are meaningfully engaged in the research process, identify the structural and intersectional barriers to authentic participation, and explore the gaps between procedural compliance and impact-driven co-design.

## 2. Materials and Methods

### 2.1. Design

Following established precedents for integrative reviews of vulnerable populations in healthcare [15,16,17,18,19], this study adopted an integrative design to synthesise heterogeneous empirical and theoretical literature, utilising PRISMA 2020 guidelines strictly as a reporting framework [20], with thematic analysis inspired by Malterud’s systematic text condensation [21]. This methodological combination, integrative design, PRISMA reporting, and systematic text condensation or thematic synthesis, has been successfully employed across diverse healthcare contexts [15,16,17,18,19]. The integrative review design was selected because it enables synthesis across diverse study types, including qualitative, quantitative, mixed-methods, and conceptual papers, which is essential for a topic as methodologically heterogeneous as CCI in migrant health research. The review protocol was prospectively registered on the Open Science Framework (Available online: https://osf.io/9v4t3 (accessed on 9 May 2026)).

### 2.2. Literature Search

**Figure 1:**
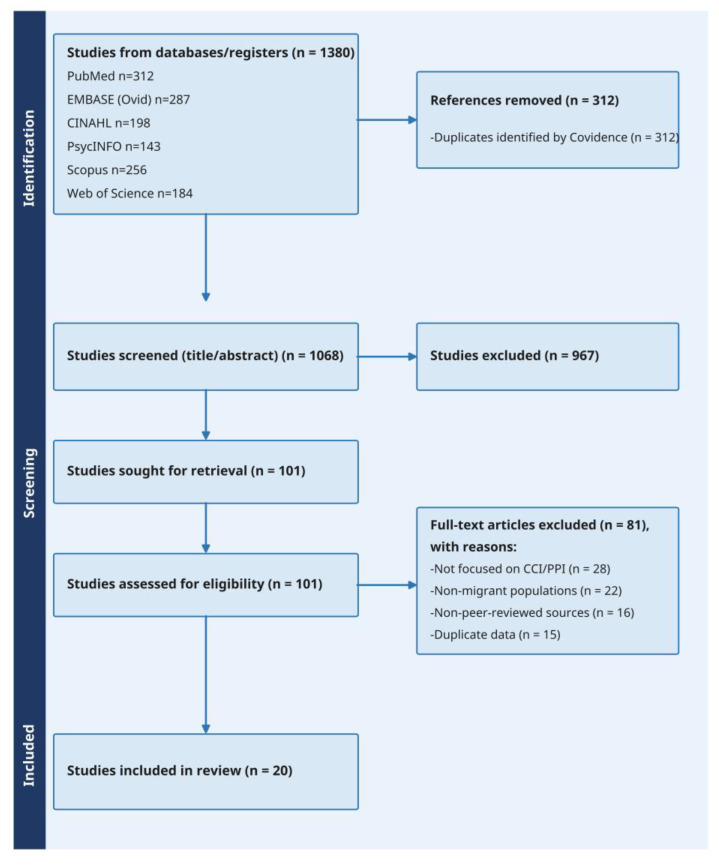
PRISMA flow diagram of study selection process.

A broad definition of “migrant” was applied to encompass CALD populations, refugees, and asylum seekers, ensuring alignment with global health equity frameworks. The search was conducted across six databases, CINAHL, EMBASE, PubMed, PsycINFO, Scopus, and Web of Science, from database inception to 15 January 2026 (full search strings available in Supplementary Table S3). Developed in collaboration with a research librarian, the search strategy was structured around three conceptual blocks: (1) Consumer and Community Involvement (CCI) or Patient and Public Involvement (PPI); (2) migrant, refugee, or CALD populations; and (3) health research contexts for minority populations. Controlled vocabulary (e.g., MeSH terms) and free-text terms were combined using Boolean operators (AND/OR), with strategies adapted to each database’s indexing system. The study selection process is illustrated in the PRISMA flow diagram (Figure 1).

### 2.3. Inclusion and Exclusion Criteria

Studies were included if they: (1) primarily involved migrant, refugee, or CALD populations, or provided conceptual, theoretical, or empirical frameworks on minority or marginalised populations with applicability to migrant health research contexts; (2) were peer-reviewed; and (3) addressed the processes, impacts, or barriers of CCI within health research governance, co-design, or participatory frameworks. Only English-language articles were eligible. Studies were excluded if they focused solely on clinical outcomes without addressing community involvement methodology, or were non-peer-reviewed. Grey literature, community reports, and policy documents were excluded. Where studies examined minority or racialised populations more broadly rather than migrant-specific groups, this is explicitly noted in results and in the relevant thematic sections.

### 2.4. Data Extraction and Quality Assessment

Two reviewers (MN and MW) independently screened titles and abstracts, followed by full-text review against predefined criteria. Disagreements were resolved through discussion with the third senior reviewer. Consensus was reached through structured discussion, consistent with the integrative review methodology adopted [15]. The review process was managed using Covidence (Veritas Health Innovation, Melbourne, Australia; available at: https://www.covidence.org/ (accessed on 9 May 2026)), which facilitated deduplication, screening, and data extraction via a standardised template.

Methodological quality was assessed using tools appropriate to each study design: AMSTAR 2 for systematic reviews [22], the Mixed Methods Appraisal Tool (MMAT) for empirical studies [23] and the JBI Critical Appraisal Checklist for Text and Opinion for conceptual papers [24]. Quality appraisal informed the descriptive context of the findings; lower-quality studies were not used as the sole basis for major analytical claims. No studies were excluded on quality grounds alone (see Supplementary Table S4). Data extracted included: country/context, population, study design, type and stage of CCI, key findings, and quality rating.

### 2.5. Analysis

Inspired by Malterud’s systematic text condensation [21], a four-step thematic analysis was applied: (1) identifying meaning units across the included texts; (2) coding meaning units into preliminary categories; (3) condensing categories into subthemes; and (4) synthesising subthemes into overarching themes. Published findings, including qualitative narratives, quantitative impact measures, and theoretical arguments, were treated as the primary data material. Analyses were conducted iteratively by MN and MW, with interpretations audited by SZ to ensure investigator triangulation and mitigate subjective bias. This hybrid approach was deductively informed by concepts of tokenism and intersectionality introduced in the literature but inductively grounded in the extracted data. The adaptation of systematic text condensation to secondary data is acknowledged as introducing an additional layer of interpretation; investigator triangulation was the primary safeguard against overinterpretation.

All authors have prior research experience in migrant health, which informed the interpretive approach taken in this review. Authors MN and SZ are themselves migrants to Australia and are actively involved in research projects with migrant and CALD communities in Australian health research contexts. To mitigate personal bias, title/abstract and full-text screening were conducted independently by two reviewers (MN and MW), and all analytical interpretations were audited by SZ. Interpretive choices are made transparent through the methods and Supplementary Materials provided.

## 3. Results

A total of 20 peer-reviewed articles were included. Included studies spanned publication years from 2003 to 2025 and employed heterogeneous designs, encompassing qualitative studies, systematic and scoping reviews, mixed-methods investigations, concept analyses, and quantitative perspectives [25,26,27,28,29,30,31,32,33,34,35,36,37,38,39,40,41,42,43,44]. Geographically, the literature drew predominantly from high-income, English-speaking countries including Australia, the United Kingdom, the United States, Canada, and New Zealand. While most studies addressed patient and public involvement or community engagement broadly, findings were critically appraised for relevance to migrant health research specifically [25,26,27,28,29,30,31,32,33,34,35,36,37,38,39,40,41,42,43,44]. Table 1 provides a summary of the 20 included studies, including country, population, study design, CCI type/stage, key findings, and quality rating.

Through systematic text condensation, four overarching themes were identified: (1) the meaningfulness gap—between procedural compliance and impact-driven involvement; (2) the burden of representation—few individuals expected to speak for heterogeneous populations; (3) intersectional power dynamics—participation skewed toward English-speaking, high-socioeconomic status migrants; and (4) limited co-design in research governance—insufficient involvement in funding, ethics, or data decisions.

### 3.1. The Meaningfulness Gap: Between Procedural Compliance and Impact-Driven Involvement

Among the included studies focusing on migrant and refugee populations, several documented involvement practices that functioned primarily as procedural compliance rather than genuine engagement. Hahn et al. (2017) [27] found that researchers often engaged communities primarily to satisfy funding mandates rather than to redistribute power or build community capacity. Similarly, Rustage et al. (2021) [41] documented that while migrants are occasionally enlisted in data collection, meaningful participation in upstream processes, including research design, priority setting, and governance, remains limited. Brammall et al. (2025) [43] further demonstrated that CCI may be appended to research as an afterthought rather than embedded from inception, which can limit its capacity to shape research direction or outcomes.

To theorise these patterns, Majid (2020) [25] offers a four-dimensional framework of tokenism (encompassing unequal power, limited impact, ulterior motives, and the antithesis of meaningful engagement) that maps closely onto the empirical conditions described above. Ocloo and Matthews (2016) [26] extend this, arguing that prevailing involvement frameworks may be structurally narrow and risk reproducing existing power hierarchies rather than disrupting them.

The contrast between current practice and demonstrated potential is instructive. Brett et al. (2014) [29] show that when involvement is enacted with genuine intent and adequate resourcing, it produces measurable benefits including improved research quality and stronger community trust. Cook et al. (2019) [28] add that realising such outcomes in marginalised settings requires flexible, culturally responsive approaches rather than rigid compliance checklists.

Taken together, these findings reveal a gap between the institutionalised rhetoric of CCI and its application in migrant health research, one that appears characterised by performative accountability over genuine community empowerment, and that may set the conditions for the burdens and exclusions examined in subsequent themes.

### 3.2. The Burden of Representation—Few Individuals Expected to Speak for Heterogeneous Populations

Among the included studies, several document the unrealistic expectation that a small number of individuals can authentically represent the full diversity of migrant communities. These individuals occupy distinct roles, including community leaders, community researchers, advisory board members, translators, and cultural brokers, each carrying different levels of authority and burden. Translators and cultural brokers mediate language and cultural access but are rarely positioned to influence research design; community researchers bear the competing demands of advocacy and academic collaboration simultaneously; advisory board members may hold nominal governance status without meaningful decision-making authority. Niemann’s (2003) [31] foundational work on the psychology of tokenism establishes the theoretical basis for understanding this strain, demonstrating that minority individuals positioned as sole community voices experience profound emotional and identity-related pressure.

Hearn et al. (2022) [30] ground this theoretically established pattern in migrant and refugee contexts specifically, documenting that community researchers from these backgrounds often navigate considerable emotional labour as they move between community advocacy and academic collaboration, roles that carry fundamentally different expectations, loyalties, and accountability structures. The structural conditions enabling this burden are further examined by Hanza et al. (2016) [33], who found that over-reliance on a small number of community gatekeepers concentrates representational responsibility and risks producing a skewed and unrepresentative picture of community perspectives.

While community gatekeepers are necessary for trust-building, safety, and access, particularly in communities with historical reasons to distrust research institutions, the problem lies not in their existence but in their over-utilisation as a substitute for broader structural inclusion. Harrison et al. (2020) [34] argue that genuine inclusion of culturally and linguistically diverse consumers demands far more than linguistic translation; it requires the deliberate cultivation of broad-based relationships that distribute representational responsibilities across a wider and more diverse network of community members. Albert and Laberge (2017) [32] offer a structural parallel from an adjacent institutional context, suggesting that confining individuals to tokenistic roles may limit their capacity to enact meaningful change, potentially transforming what should be a mechanism for equity into an administrative burden that serves institutional interests rather than community ones.

Collectively, these findings suggest a recurring pattern of inadequacy in how CCI frameworks conceptualise representation in migrant health research. Rather than designing for diversity and distributing participation broadly, current approaches frequently concentrate responsibility on the few amplifying individual burden while obscuring the structural inequities that make heterogeneous, authentic representation so difficult to achieve.

### 3.3. Intersectional Power Dynamics—Participation Skewed Toward English-Speaking, Higher-Socioeconomic Status Migrants

It is noted that some studies contributing to this theme examine minority or racialised populations broadly rather than migrant populations specifically. Their findings are included where the structural dynamics identified are directly applicable to migrant health research contexts and are distinguished from migrant-specific evidence accordingly. Since participant demographic data were often poorly reported in the primary studies, the findings below should be interpreted as observed trends rather than definitive quantitative conclusions.

Abrams et al. (2020) [35] and Muirhead et al. (2020) [36], drawing on minority population samples, demonstrate that overlapping identities, including race, gender, language proficiency, and socioeconomic status, compound one another to produce layered forms of marginalisation in health research participation. While these studies do not focus exclusively on migrant populations, the intersectional dynamics they identify have clear structural relevance to migrant communities, where language barriers, precarious legal status, and socioeconomic disadvantage frequently converge.

The practical consequences of these dynamics in migrant and minority health research contexts are examined by George et al. (2014) [38], whose systematic review found that structural and linguistic obstacles disproportionately exclude non-English speakers and those of lower socioeconomic standing from research participation. Applied to migrant health research, this suggests that CCI initiatives may default toward engaging migrants who are already more assimilated and more resourced, and therefore less representative of the communities most affected by health inequity. Critically, this is not a matter of individual reluctance. Wendler et al. (2006) [39], studying minority populations broadly, challenge the persistent but empirically unsupported assumption that minorities are disinclined to participate in research, demonstrating quantitatively that structural barriers, not attitudinal deficits, drive exclusion. While Wendler et al. do not examine migrant populations specifically, their findings directly counter deficit-based explanations that risk misattributing structural failures to community unwillingness.

Agénor (2020) [37] sharpens this critique at the level of research design, arguing that without an explicitly intersectional framework, population health studies risk homogenising migrants into a single undifferentiated category, potentially obscuring the distinct needs of the most marginalised subgroups. MacFarlane et al. (2024) [40], examining refugee and migrant populations in European contexts specifically, extend this to governance, calling for migrant voices to be embedded at the highest levels of research agenda-setting rather than consulted selectively at the margins.

Together, these findings drawn from both minority population research and migrant-specific studies, suggest a possible paradox at the core of CCI practice: the communities most affected by health disparities may be the least likely to be meaningfully included in the research designed to address them. Addressing this requires not merely widening recruitment nets but rethinking the power structures that determine who is invited to participate, on whose terms, and toward whose benefit.

### 3.4. Limited Co-Design in Research Governance—Rarely Present Migrant Voices in Funding, Ethics, and Data Decisions

The included studies expose the limited involvement of migrant communities across three distinct levels of research governance: (1) research design and priority setting; (2) ethics review and oversight; and (3) data governance. Each level reflects a different dimension of exclusion, and the evidence across them is uneven, a pattern that is itself a finding of this review.

The most consistently evidenced governance gap concerns exclusion from upstream research processes. Rustage et al. (2021) [41] document that while migrants are occasionally enlisted in data collection, their meaningful participation in research design and priority setting is rarely reported in the reviewed literature. MacFarlane et al. (2024) [40], examining refugee and migrant populations in European contexts, call for a paradigm shift that normalises participatory research as a baseline requirement, with migrant voices embedded at the level of agenda-setting rather than consulted selectively at later stages. Brammall et al. (2025) [43] provide empirical support for this position in an Australian CALD context, demonstrating that embedding CCI from inception, rather than appending it as a methodological afterthought, is a prerequisite for developing genuinely culturally competent research and care.

No included study directly examined migrant community involvement in ethics review processes. This absence is itself significant: if migrant communities are not involved in determining what constitutes ethical research practice in their own contexts, the ethical frameworks governing that research risk reflecting institutional rather than community values. This represents an identified gap in the primary literature warranting dedicated future investigation.

Miller et al. (2018) propose a comprehensive governance framework arguing that CCI must be reconceptualised as a systemic policy imperative rather than a discretionary methodological choice, one that extends to decisions about how community data are collected, owned, and used [42]. The question of who exercises meaningful control over health data generated from migrant communities remains largely unaddressed in the included studies. While scholarship on Indigenous data sovereignty, most notably Kukutai and Taylor (2016) [44], has developed robust frameworks for community data control, the direct transfer of these frameworks to migrant populations requires careful theoretical adaptation that falls outside the scope of this review. The applicability of data sovereignty principles to migrant health research nonetheless represents a significant and underexplored area for future work.

Taken together, these findings suggest that co-design in research governance is insufficiently evidenced at most levels examined. The rhetoric of community involvement appears to have been broadly institutionalised, yet the reviewed literature suggests that migrant communities remain insufficiently represented from the decisions that most fundamentally shape the research purportedly conducted in their interest, decisions about what is studied, what is considered ethical, and who owns the resulting knowledge.

## 4. Discussion

This integrative review critically examined the depth of Consumer and Community Involvement in migrant health research across 20 peer-reviewed studies. The findings reveal a substantive and persistent disjuncture between prevailing policy commitments to inclusive research and the empirical reality of engagement. Four overarching themes emerged: the adequacy of current involvement practices, the inequitable distribution of representational burden, the influence of intersectional power dynamics, and the extent to which migrant communities are included in research governance structures. Considered together, these themes suggest that CCI in the reviewed migrant health research remains a predominantly marginal enterprise, shaped by structural conditions that appear to reproduce exclusion across multiple levels.

The persistence of tokenistic involvement despite widespread policy mandates for meaningful CCI points to a structural rather than an attitudinal problem. Researchers, ethics committees, and funding bodies have broadly adopted the language of community involvement, yet the reviewed studies suggest this has not translated into redistribution of epistemic authority or decision-making power. The gap between rhetoric and practice is not incidental, it reflects institutional incentive structures that reward compliance with involvement requirements over investment in the slower, more resource-intensive work of genuine co-design. Hahn et al. (2017) document this dynamic directly, finding that community engagement is frequently driven by funding mandates rather than authentic commitment to power redistribution [27]. This suggests that reform cannot be achieved through researcher behaviour alone; it requires structural change at the level of how involvement is defined, resourced, and evaluated by the bodies that govern research.

The themes of representational burden and intersectional power dynamics together challenge a foundational assumption embedded in current CCI practice, that migrant communities can be treated as a coherent or homogeneous group. Engagement strategies across the reviewed literature often appeared to default to migrants who were educated, English-speaking, or socioeconomically advantaged, suggesting that the most vulnerable subgroups, including undocumented individuals, newly arrived refugees, and those with limited English proficiency, may be insufficiently represented in the research process [34,37]. Consistent with Wendler et al. (2006), this pattern is better understood as a structural failure of research design than as evidence of community disengagement [39]. The implication is that intersectional recruitment frameworks, designed from the outset to reach those most marginalised, are not a methodological refinement but a prerequisite for research that claims to serve migrant communities.

The nature of involvement across the reviewed studies further reveals a well-defined hierarchy of participation. Migrant involvement was frequently reported as confined to later, consultative phases, data collection, linguistic translation, and intervention validation, rather than the formative processes of priority setting, funding deliberation, and study design [36,37]. This pattern suggests an insufficient recognition of migrants as legitimate epistemic partners with authoritative standing over research agendas that purport to serve their communities. MacFarlane et al. (2024) argue that normalising participatory research as a baseline requirement, with migrant voices embedded at the level of governance and agenda-setting, represents the necessary direction of reform [40]. Miller et al. (2018) extend this to policy, proposing that CCI be reconceptualised as a systemic imperative rather than a discretionary methodological choice [42]. Taken together, these positions suggest that meaningful reform requires governance-level intervention: in how ethics committees evaluate involvement plans, in how funding bodies weight co-design in grant assessment, and in how research institutions build and sustain long-term community partnerships.

As digital health infrastructures expand, there is a risk that the structural inequities identified in this review are reproduced in new domains [45]. Emerging scholarship, outside the scope of the included studies but relevant as contextual literature, highlights the limited evidence base for digital tools in migrant and refugee health, and the risks of widening inequity where migrants are excluded from the design, implementation, and evaluation of those tools [46]. Questions of data governance in digital contexts, who exercises control over health data, under what conditions, and toward whose benefit, extend the governance concerns identified in Theme 3.4 into an emerging and underregulated domain. These questions warrant dedicated empirical investigation in future research.

Drawing the four themes together, a coherent picture emerges: CCI in migrant health research appears constrained not by a lack of policy commitment but by structural conditions that may reproduce exclusion, concentrated representational burden, intersectional participation barriers, and insufficiently evidenced governance-level co-design. Addressing these conditions requires moving beyond procedural compliance toward reform that embeds migrant communities as epistemic partners across the full research cycle, from priority setting and funding decisions to ethics oversight and data governance. Future research should develop and empirically evaluate intersectional recruitment frameworks and migrant-led co-design models specifically designed to reach and engage the most marginalised migrant subgroups.

### 4.1. Implications for Practice and Policy

The findings carry significant and concrete implications for researchers, ethics committees, and funding bodies alike. Procedural compliance, appointing a community representative to an advisory board or ticking a consumer involvement box on a grant application, appears insufficient on its own. Funding bodies must go further: mandating and financially resourcing the relationship-building, trust development, and iterative co-design processes that must precede the finalisation of any research question. Ethics committees, in turn, should require applicants to explicitly demonstrate how intersectional power dynamics will be identified and addressed, and how the burden of representation will be actively distributed rather than concentrated on a handful of community liaisons.

For researchers, the imperative is equally clear. Flexible, culturally safe methodologies are needed to supplement or replace the gatekeeping recruitment models that appear to dominate practice. This means actively cultivating broad-based community networks rather than relying on singular community leaders or translators; providing meaningful and fair remuneration for community involvement as standard; offering professional translation and interpretation as a recommended baseline; and ensuring that research outputs are communicated back to participating communities in accessible, culturally appropriate formats. Authentic partnership cannot be an afterthought, it must be architecturally embedded from the outset.

### 4.2. Limitations and Future Directions

Despite the contributions this review makes to the field, some limitations warrant acknowledgement. First, the restriction to English-language, peer-reviewed publications excluded grey literature, community reports, and studies in other languages, likely biassing the review toward institutionally mediated accounts in high-income Anglophone countries and obscuring non-Western participatory paradigms. Second, the heterogeneity of study designs required interpretive judgement during synthesis; findings should therefore be considered as thematic trends rather than statistically generalisable conclusions. Third, there is conceptual slippage across the included literature between the terms “migrant,” “refugee,” “CALD,” and “ethnic minority,” which may affect the precision of the synthesis. Fourth, the geographic concentration in high-income countries limits the transferability of findings to other migrant health systems. This geographic concentration is itself analytically significant: the absence of CCI evidence from non-Anglophone contexts may reflect the uneven global distribution of research infrastructure, suggesting that structural inequities in participation may extend to knowledge production itself.

Future research must orient itself toward structural intervention rather than descriptive critique. To support this reorientation, Table 2 presents an evidence-grounded framework that maps each of the four identified themes to its corresponding structural barrier, reform direction, and the governance level at which change is required. The framework is intended not as a prescriptive checklist but as a scaffold for translating the thematic findings of this review into actionable research and policy priorities, moving along a continuum from procedural compliance toward governance-level co-design and, ultimately, authentic migrant partnership. Rigorous empirical evaluation of co-design models embedded at the level of research governance, including funding allocation, ethics review, and data sovereignty frameworks, is a priority for future research. Studies specifically designed to test intersectional recruitment strategies capable of reaching the most marginalised migrant subgroups, including non-English speakers and undocumented individuals, represent a critical and largely unaddressed gap in the current evidence base. Longitudinal research examining the sustained impact of migrant-led data sovereignty initiatives on both research quality and health outcomes would also make a meaningful contribution. Ultimately, the most important reorientation the field can make is epistemic: to stop asking why migrants are not participating, and to start asking how research structures might be reformed to make authentic, power-sharing partnership not merely possible, but a realistic and sustained goal.

## 5. Conclusions

CCI in migrant health research remains more procedural than transformative. The four themes identified in this review, the meaningfulness gap, the burden of representation, intersectional power dynamics, and limited co-design in research governance, converge on a single structural diagnosis: that authentic community partnership is not failing because of individual researcher shortcomings, but because the institutional conditions governing research are not designed to produce it. Procedural compliance appears to have been prioritised over equitable participation; the latter remains insufficiently evidenced across the reviewed literature. Closing this gap demands systemic reform across funding mechanisms, ethics oversight, and research governance, reform oriented not toward better managing migrant involvement, but toward recognising migrant communities as legitimate epistemic partners with authoritative standing over the research agendas that shape their health.

## Figures and Tables

**Table 1:** Summary of 20 included studies.

Theme	Reference	Country	Population	Study Design/ Evidence Type	CCI Type/Stage	Key Finding	Quality Rating
1. The Meaningfulness Gap	Majid (2020) [25]	Canada	General/PPI	Concept Analysis/Theoretical	Involvement design	Identified four dimensions of tokenism: unequal power, limited impact, ulterior motives, and the antithesis of meaningful engagement.	High
	Ocloo & Matthews (2016) [26]	UK	General/PPI	Conceptual Perspective/Theoretical	Governance	Argued that current PPI frameworks are structurally narrow and exclusionary, reproducing tokenism and leaving existing power hierarchies intact.	High
	Hahn et al. (2017) [27]	USA	General/PPI	Qualitative/Empirical	Funding/Design	Found that researchers routinely engage communities to satisfy funding mandates rather than to genuinely redistribute power or build community capacity.	Moderate
	Cook et al. (2019) [28]	Multiple (LMICs)	Minority/LMIC	Systematic Review/Empirical	All stages	Highlighted the need for flexible, culturally adapted approaches to move beyond tokenistic compliance checklists in Low- and Middle-Income Country.	High
	Brett et al. (2014) [29]	UK	General/PPI	Systematic Review/Empirical	All stages	Demonstrated that when PPI is enacted with genuine intent and adequate resourcing, it generates measurable benefits including improved research quality and enhanced community trust.	High
2. Burden of Representation	Hearn et al. (2022) [30]	Australia	Refugee/Migrant	Qualitative/Empirical	Community researcher role	Revealed the intense emotional labour and competing demands faced by community researchers from refugee and migrant backgrounds acting as both advocates and academic collaborators.	High
	Niemann (2003) [31]	USA	Minority populations	Book Chapter/Conceptual and Theoretical	Representation	Established the foundational psychology of tokenism, demonstrating the profound emotional and identity-related strain placed on minority individuals positioned as sole community voices.	High
	Albert & Laberge (2017) [32]	Canada	Social scientists	Qualitative/Empirical	Governance/Institutional roles	Showed that confining individuals to tokenistic institutional roles neutralises their capacity to enact systemic change, transforming a mechanism for equity into an administrative burden.	Moderate
	Hanza et al. (2016) [33]	USA	Immigrant/Refugee	Mixed Methods/ Empirical	Recruitment/Gatekeeping	Identified that over-reliance on a small number of community gatekeepers concentrates the representational burden and risks producing a skewed picture of community perspectives.	High
	Harrison et al. (2020) [34]	Australia	CALD populations/Migrants	Qualitative/Empirical	Community engagement	Argued that genuine inclusion of CALD consumers requires the deliberate cultivation of broad-based relationships that distribute representational responsibilities across a wider, more diverse network.	Moderate
3. Intersectional Power Dynamics	Abrams et al. (2020) [35]	USA	Minority populations	Methodological/Conceptual and Theoretical	Research design	Emphasised the necessity of applying intersectionality theory to understand how overlapping identities, race, gender, language, socioeconomic status, compound marginalisation in health research.	High
	Muirhead et al. (2020) [36]	UK	Minority populations	Perspective/Review / Empirical	Research design	Discussed how intersectional power dynamics may limit participation of the most vulnerable subgroups in oral health and broader health research.	Moderate
	Agénor (2020) [37]	USA	Minority populations	Quantitative Perspective/Conceptual and Theoretical	Research design	Argued that without an explicitly intersectional framework, quantitative health research masks power dynamics and homogenises migrants into a single category.	High
	George et al. (2014) [38]	USA	Minority populations	Systematic Review/Empirical	Participation barriers	Found that structural and linguistic obstacles disproportionately exclude non-English speakers and those of lower socioeconomic status from health research participation.	High
	Wendler et al. (2006) [39]	USA	Minority populations	Quantitative/Empirical	Participation barriers	Challenged the assumption that minorities are unwilling to participate, demonstrating quantitatively that structural barriers, not attitudinal deficits, drive exclusion.	Moderate
4. Limited Co-Design in Research Governance	MacFarlane et al. (2024) [40]	Europe	Refugee/Migrant	Narrative Synthesis/Position Paper/Empirical	Governance/Priority setting	Called for a fundamental paradigm shift that normalises participatory research as a baseline requirement, with migrant voices embedded at the highest levels of governance and agenda-setting.	High
	Rustage et al. (2021) [41]	Multiple	Migrant populations	Systematic Review/Empirical	Intervention design/Governance	Documented that while migrants are occasionally enlisted in data collection, meaningful participation in upstream processes of research design, priority setting, and governance is rarely reported.	High
	Miller et al. (2018) [42]	Multiple	General/PPI	Scoping Review + Framework Development/Empirical	Governance/Policy	Proposed a comprehensive governance framework arguing that CCI must be reconceptualised as a systemic policy imperative rather than a discretionary methodological choice.	High
	Brammall et al. (2025) [43]	Australia	CALD populations	Mixed Methods/Empirical	Research design/Co-design	Demonstrated that embedding CCI directly into research methodology from inception, rather than as an afterthought, is a prerequisite for developing genuinely culturally competent care.	High
	Kukutai & Taylor (2016) [44] *	New Zealand/Australia	Indigenous populations	Edited Book/Contextual/Theoretical Literature	Data governance/Sovereignty	Articulated the ethical imperative for marginalised communities to exercise meaningful control over their own health data. Included as contextual theoretical literature.	High

CCI = Consumer and Community Involvement; PPI = Patient and Public Involvement; CALD = Culturally and Linguistically Diverse; LMIC = Low- and Middle-Income Country. Quality ratings: High = low risk of bias; Moderate = some methodological limitations. Quality appraisal tools: AMSTAR 2 (systematic reviews); MMAT (empirical studies); JBI Critical Appraisal Checklist for Text and Opinion (conceptual papers). * Kukutai & Taylor (2016) [44] is included as contextual theoretical literature only; direct transfer of Indigenous data sovereignty frameworks to migrant populations requires careful adaptation beyond the scope of this review.

**Table 2:** Evidence-grounded framework for future directions in Consumer and Community Involvement (CCI) for migrant health research.

Evidence-Grounded Framework for Future Directions in CCI for Migrant Health Research				
Theme Header	The Meaningfulness Gap	Burden of Representation	Intersectional Power Dynamics	Limited Co-Design in Research Governance
Structural barrier identified	CCI functions as procedural compliance rather than impact-driven partnership. Gatekeeping by institutions may limit authentic migrant voice.	Few individuals expected to represent heterogeneous migrant communities; roles concentrate emotional and administrative burden unfairly.	Participation skewed toward English-speaking, higher-SES migrants; most marginalised subgroups appear insufficiently represented.	Migrants rarely present in funding, ethics, and data decisions; upstream involvement is rarely reported and appears structurally limited.
Evidence- informed reform direction	Mandate governance-level co-design from research inception; require funders to resource iterative and sustained community engagement beyond single consultation events.	Develop distributed representation models; provide fair remuneration and professional support for community researchers; build broad-based advisory networks.	Adopt intersectional recruitment frameworks; provide multilingual access and interpreter support; apply intersectionality as an analytic method in future reviews.	Embed migrant voices in priority setting, funding allocation, and data governance; extend to digital health infrastructures and data sovereignty frameworks.
Governance level where reform is required	Funding bodies & Research institutions	Research teams & Ethics committees	Research design & Ethics review	Funding bodies, Ethics committees & Data governance bodies
Reform continuum: Procedural Compliance → Governance-Level Co-Design → Authentic Migrant Partnership				

Note: All reform directions are grounded in the 20 studies included in this review. Arrows indicate a logical reform sequence, not empirically established causal pathways [25,26,27,28,29,30,31,32,33,34,35,36,37,38,39,40,41,42,43,44].

## Data Availability

No new data were created or analysed in this study. Data sharing is not applicable to this article.

## Supplementary Materials

The following supporting information can be downloaded at: https://www.mdpi.com/article/10.3390/healthcare14142065/s1, Table S1: PRISMA 2020 Abstract Checklist; Table S2: PRISMA 2020 Checklist; Table S3: Database-Specific Search Strings; Table S4: Quality Assessment.
