# Supplementary material for: Meaningful or Marginal: An Integrative Review of Consumer and Community Involvement in Migrant Health Research
Source: Healthcare (Basel). 2026 Jul 9;14(14):2065. doi: 10.3390/healthcare14142065 (PMC13409862; doi:10.3390/healthcare14142065)
Supplement: Supplementary file 1 [file healthcare-14-02065-s001.zip › Revised_TableS3_Database-SpecificSearchStrings.pdf]

Table S3: Database-Specific Search Strings

The following search strings were adapted to each database's controlled vocabulary and indexing conventions. All searches were conducted from database inception to January 2026.

PubMed	(("Consumer Participation"[MeSH] OR "Patient Participation"[MeSH] OR "Community Participation"[MeSH] OR "consumer involvement" OR "community involvement" OR "patient and public involvement" OR "co-design" OR "co-production" OR "participatory research" OR "lived experience" OR "stakeholder engagement" OR "lay involvement")) AND (("Emigrants and Immigrants"[MeSH] OR "Refugees"[MeSH] OR "Transients and Migrants"[MeSH] OR "migrant" OR "immigrant" OR "refugee" OR "asylum seeker" OR "CALD" OR "culturally and linguistically diverse" OR "ethnic minority" OR "foreign-born")) AND (("Health Research"[MeSH] OR "Biomedical Research"[MeSH] OR "Public Health"[MeSH] OR "health research" OR "medical research" OR "clinical research" OR "research governance" OR "health intervention" OR "evidence-based practice"))
**EMBASE (via Ovid)**	('consumer involvement' OR 'patient and public involvement' OR 'community involvement' OR 'co-design' OR 'co-production' OR 'participatory research' OR 'lived experience' OR 'stakeholder engagement' OR 'community participation'/exp) AND ('migrant' OR 'immigrant' OR 'refugee' OR 'asylum seeker' OR 'CALD' OR 'culturally and linguistically diverse' OR 'ethnic minority' OR 'foreign born' OR 'emigrant'/exp OR 'refugee'/exp) AND ('health research' OR 'medical research' OR 'clinical research' OR 'public health'/exp OR 'research governance' OR 'health intervention' OR 'evidence based practice')
**CINAHL (via EBSCOhost)**	(MH "Consumer Participation" OR MH "Community Participation" OR MH "Patient Participation" OR TI "consumer involvement" OR AB "consumer involvement" OR TI "patient and public involvement" OR AB "patient and public involvement" OR TI "co-design" OR TI "co-production" OR TI "participatory research" OR TI "lived experience" OR TI "stakeholder engagement")

	AND<br>(MH "Immigrants" OR MH "Refugees" OR MH "Transients and Migrants"<br>OR TI "migrant" OR TI "immigrant" OR TI "refugee" OR TI "asylum seeker"<br>OR TI "CALD" OR TI "culturally and linguistically diverse" OR TI "ethnic minority")<br>AND<br>(MH "Health Research" OR MH "Public Health" OR TI "health research"<br>OR TI "medical research" OR TI "research governance" OR TI "health intervention")
**PsycINFO (via APA PsycNet / Ovid)**	(DE "Community Involvement" OR DE "Participatory Action Research" OR DE "Patient Participation"<br>OR "consumer involvement" OR "patient and public involvement" OR "co-design"<br>OR "co-production" OR "participatory research" OR "lived experience"<br>OR "stakeholder engagement" OR "lay involvement")<br>AND<br>(DE "Immigrants" OR DE "Refugees" OR DE "Cultural Diversity"<br>OR "migrant" OR "immigrant" OR "refugee" OR "asylum seeker" OR "CALD"<br>OR "culturally and linguistically diverse" OR "ethnic minority" OR "foreign-born")<br>AND<br>("health research" OR "medical research" OR "clinical research" OR "public health"<br>OR "research governance" OR "health intervention" OR "evidence-based practice")
**Scopus**	TITLE-ABS-KEY<br>(("consumer involvement" OR "community involvement" OR "patient and public involvement"<br>OR "co-design" OR "co-production" OR "participatory research" OR "lived experience"<br>OR "stakeholder engagement" OR "lay involvement")<br>AND<br>("migrant" OR "immigrant" OR "refugee" OR "asylum seeker" OR "CALD"<br>OR "culturally and linguistically diverse" OR "ethnic minority" OR "foreign-born")<br>AND<br>("health research" OR "medical research" OR "clinical research" OR "public health"<br>OR "research governance" OR "health intervention" OR "evidence-based practice")<br>)
**Web of Science (Core Collection)**	TS=<br>(("consumer involvement" OR "community involvement" OR "patient and public involvement"<br>OR "co-design" OR "co-production" OR "participatory

	research" OR "lived experience"<br>OR "stakeholder engagement" OR "lay involvement")<br>AND<br>("migrant" OR "immigrant" OR "refugee" OR "asylum seeker" OR "CALD"<br>OR "culturally and linguistically diverse" OR "ethnic minority" OR "foreign-born")<br>AND<br>("health research" OR "medical research" OR "clinical research" OR "public health"<br>OR "research governance" OR "health intervention" OR "evidence-based practice")<br>)

Boolean Logic Structure

```
[ Block 1: CCI / PPI Terms ]
           AND
[ Block 2: Migrant / CALD / Refugee Terms ]
           AND
[ Block 3: Health Research Context Terms ]

Within each block: terms combined with  OR
Between blocks:    blocks combined with AND
```

Note: Search strategies were adapted to each database's controlled vocabulary and indexing conventions. All searches were conducted from database inception to January 2026, in collaboration with a research librarian. The PRISMA 2020 flow diagram illustrating the full study selection process is presented as Figure 1 in the main manuscript.
