# Supplementary material for: Meaningful or Marginal: An Integrative Review of Consumer and Community Involvement in Migrant Health Research
Source: Healthcare (Basel). 2026 Jul 9;14(14):2065. doi: 10.3390/healthcare14142065 (PMC13409862; doi:10.3390/healthcare14142065)
Supplement: Supplementary file 1 [file healthcare-14-02065-s001.zip › Table S4_Quality Assessment .pdf]

Table S4: Quality Assessment

The following table presents the quality assessment of the 20 peer-reviewed studies included in the integrative review. The appraisal tools used were selected based on the study design:

- **AMSTAR 2** for systematic reviews (rated as High, Moderate, Low, or Critically Low confidence) [22].
- **MMAT (Mixed Methods Appraisal Tool) 2018** for qualitative, quantitative, and mixed methods empirical studies (rated using the recommended percentage score: 20%, 40%, 60%, 80%, or 100% based on the number of criteria met, replacing the previous star rating system) [23].
- **The JBI Critical Appraisal Checklist** for Text and Opinion for conceptual papers [24].

#	Author (Year)	Study Design	Appraisal Tool	Quality Rating	Key Appraisal Notes
1	Majid (2020) [25]	Concept Analysis	Theory Critique	High	Rigorous conceptual framework; high relevance to tokenism.
2	Ocloo & Matthews (2016) [26]	Perspective/Review	Narrative Critique	High	Strong theoretical argument; highly cited in CCI literature.
3	Hahn et al. (2017) [27]	Qualitative/Perspective	MMAT (Qual)	80%	Clear methodology, though limited to a specific medical context.
4	Cook et al. (2019) [28]	Systematic Review	AMSTAR 2	High	Comprehensive search strategy and robust data synthesis.
5	Brett et al. (2014) [29]	Systematic Review	AMSTAR 2	High	Landmark study on PPI impact; excellent methodological rigour.
6	Hearn et al. (2022) [30]	Qualitative (Participatory)	MMAT (Qual)	100%	Exemplary community researcher involvement and ethical depth.
7	Niemann (2003) [31]	Book Chapter (Theory)	Narrative Critique	High	Foundational psychological theory on tokenism.
8	Albert & Laberge (2017) [32]	Qualitative	MMAT (Qual)	80%	Strong sociological framework; clear data-to-theory link.
9	Hanza et al. (2016) [33]	Mixed Methods	MMAT (Mixed)	100%	Clear rationale for mixing methods in migrant recruitment.
10	Harrison et al. (2020) [34]	Qualitative	MMAT (Qual)	80%	Relevant to CALD contexts; sound thematic analysis.
11	Abrams et al. (2020) [35]	Methodological Paper	Theory Critique	High	Critical framework for intersectionality in health research.
12	Muirhead et al. (2020) [36]	Perspective/Review	Narrative Critique	Moderate	Focused on oral health, but intersectionality theory is sound.

13	Agénor (2020) [37]	Quantitative Perspective	Theory Critique	High	Essential critique of quantitative power dynamics.
14	George et al. (2014) [38]	Systematic Review	AMSTAR 2	High	Robust analysis of barriers across multiple ethnicities.
15	Wendler et al. (2006) [39]	Quantitative (Survey)	MMAT (Quant)	80%	Significant sample size; challenges prevailing assumptions.
16	MacFarlane et al. (2024) [40]	Policy/Paradigm Shift	Narrative Critique	High	WHO-backed; highly authoritative on migrant governance.
17	Rustage et al. (2021) [41]	Systematic Review	AMSTAR 2	High	Recent and comprehensive look at migrant interventions.
18	Miller et al. (2018) [42]	Governance Framework	Theory Critique	High	Comprehensive systemic model for research involvement.
19	Brammall et al. (2025) [43]	Qualitative/Methodology	MMAT (Qual)	100%	Up-to-date; high relevance to Australian CALD populations.
20	Kukutai & Taylor (2016) [44]	Edited Book (Theory)	Narrative Critique	High	Foundational text for data sovereignty principles.

Notes on Appraisal Tool Scoring Corrections

- **AMSTAR 2**: The previous table used a star rating system for AMSTAR 2, which is incorrect. AMSTAR 2 does not generate an overall score but rather an overall confidence rating (High, Moderate, Low, Critically Low) based on critical domains. The ratings have been corrected to reflect this.
- **MMAT 2018**: The previous table used a star rating system (e.g., ★★★★☆). The official MMAT 2018 guidelines discourage presenting an overall score, but if one is needed, it recommends using a percentage (20%, 40%, 60%, 80%, 100%) based on the number of criteria met (1 to 5). The ratings have been updated to the percentage format for accuracy.

References

22. Shea, B.J., et al., AMSTAR 2: a critical appraisal tool for systematic reviews that include randomised or non-randomised studies of healthcare interventions, or both. BMJ, 2017. 358: p. j4008.
23. Hong, Q.N., et al., The Mixed Methods Appraisal Tool (MMAT) version 2018 for information professionals and researchers. Education for Information, 2018. 34(4): p. 285–291.
24. Institute, J.B., Critical appraisal checklist for text and opinion. 2017.
25. Majid, U., The dimensions of tokenism in patient and family engagement: a concept analysis of the literature. Journal of Patient Experience, 2020. 7(6): p. 1610–1620.
26. Ocloo, J. and R. Matthews, From tokenism to empowerment: progressing patient and public involvement in healthcare improvement. BMJ Quality & Safety, 2016. 25(8): p. 626–632.
27. Hahn, D.L., et al., Tokenism in patient engagement. Family Practice, 2017. 34(3): p. 290–295.
28. Cook, N., et al., Patient and public involvement in health research in low and middle-income countries: a systematic review. BMJ Open, 2019. 9(5): p. e026514.
29. Brett, J., et al., A systematic review of the impact of patient and public involvement on service users, researchers and communities. The Patient — Patient-Centered Outcomes Research, 2014. 7(4): p. 387–395.
30. Hearn, F., et al., Having a say in research directions: the role of community researchers in participatory research with communities of refugee and migrant background. International Journal of Environmental Research and Public Health, 2022. 19(8): p. 4844.
31. Niemann, Y.F., The psychology of tokenism, in Handbook of Racial and Ethnic Minority Psychology. Sage Publications, 2003. p. 100–118.

32. Albert, M. and S. Laberge, Confined to a tokenistic status: social scientists in leadership roles in a national health research funding agency. Social Science & Medicine, 2017. 185: p. 137–146.
33. Hanza, M.M., et al., Lessons learned from community-led recruitment of immigrants and refugee participants for a randomized, community-based participatory research study. Journal of Immigrant and Minority Health, 2016. 18(5): p. 1241–1245.
34. Harrison, R., et al., Beyond translation: engaging with culturally and linguistically diverse consumers. Health Expectations, 2020. 23(1): p. 159–168.
35. Abrams, J.A., et al., Considerations for employing intersectionality in qualitative health research. Social Science & Medicine, 2020. 258: p. 113138.
36. Muirhead, V.E., et al., What is intersectionality and why is it important in oral health research? Community Dentistry and Oral Epidemiology, 2020. 48(6): p. 464–470.
37. Agénor, M., Future directions for incorporating intersectionality into quantitative population health research. American Journal of Public Health, 2020. 110(6): p. 803–806.
38. George, S., N. Duran, and K. Norris, A systematic review of barriers and facilitators to minority research participation among African Americans, Latinos, Asian Americans, and Pacific Islanders. American Journal of Public Health, 2014. 104(2): p. e16–e31.
39. Wendler, D., et al., Are racial and ethnic minorities less willing to participate in health research? PLoS Medicine, 2006. 3(2): p. e19.
40. MacFarlane, A., et al., Normalising participatory health research approaches in the WHO European region for refugee and migrant health: a paradigm shift. The Lancet Regional Health – Europe, 2024. 41: p. 100916.
41. Rustage, K., et al., Participatory approaches in the development of health interventions for migrants: a systematic review. BMJ Open, 2021. 11(10): p. e053678.
42. Miller, F.A., et al., Public involvement in health research systems: a governance framework. Health Research Policy and Systems, 2018. 16(1): p. 79.
43. Brammall, B.R., et al., Embedding consumer and community involvement in antenatal healthcare research: a methodological approach and perspectives of culturally and linguistically diverse women in Australia. Research Involvement and Engagement, 2025. 11(1): p. 41.
44. Kukutai, T. and J. Taylor, Indigenous Data Sovereignty: Toward an Agenda. Vol. 38. Canberra: ANU Press, 2016.
